# p53/*TP53* Status Assessment in Gastroesophageal Adenocarcinoma

**DOI:** 10.3390/cancers15102783

**Published:** 2023-05-16

**Authors:** Elisa Boldrin, Maria Assunta Piano, Francesco Bernaudo, Rita Alfieri, Maria Raffaella Biasin, Isabella Monia Montagner, Alice Volpato, Genny Mattara, Francesco Lamacchia, Giovanna Magni, Antonio Rosato, Antonio Scapinello, Pierluigi Pilati, Matteo Curtarello

**Affiliations:** 1 Immunology and Molecular Oncology Diagnostics Unit, Veneto Institute of Oncology IOV-IRCCS, 35128 Padova, Italy; mariaassunta.piano@iov.veneto.it (M.A.P.); francesco.bernaudo@iov.veneto.it (F.B.); antonio.rosato@iov.veneto.it (A.R.); matteo.curtarello@iov.veneto.it (M.C.); 2 Surgical Oncology of Digestive Tract Unit, Veneto Institute of Oncology IOV-IRCCS, 35128 Padova, Italy; rita.alfieri@humanitas.it (R.A.); genny.mattara@iov.veneto.it (G.M.); francesco.lamacchia@iov.veneto.it (F.L.); pierluigi.pilati@iov.veneto.it (P.P.); 3 Anatomy and Pathological Histology Unit, Veneto Institute of Oncology IOV-IRCCS, 35128 Padova, Italy; mariaraffaella.biasin@iov.veneto.it (M.R.B.); isabellamonia.montagner@iov.veneto.it (I.M.M.); alice.volpato@iov.veneto.it (A.V.); antonio.scapinello@iov.veneto.it (A.S.); 4 Department of Surgery Oncology and Gastroenterology, University of Padova, 35122 Padova, Italy; 5 Clinical Research Unit, Veneto Institute of Oncology IOV-IRCCS, 35128 Padova, Italy

**Keywords:** p53, *TP53*, gastroesophageal adenocarcinoma, chromosomal instability (CIN), liquid biopsy, cell-free DNA (cfDNA), droplet digital PCR (ddPCR), next-generation sequencing (NGS)

## Abstract

**Simple Summary:**

Despite p53 aberration, as a prognostic biomarker, still remaining a matter of debate for gastroesophageal adenocarcinoma (GEA), the characterization of p53/*TP53* is routinely performed to assign chromosomal instability (CIN). The current gold standard for p53 assessment is immunohistochemistry (IHC). However, several studies with other tumors have demonstrated that “low” IHC staining levels should be considered as aberrant as “strong” staining due to mutated p53. We investigated the potential of molecular assays, such as droplet digital PCR and next-generation sequencing, for the implementation of IHC. The results suggest that molecular approaches in solid and liquid biopsies could improve the characterization of “low” IHC cases, revealing that the majority harbor a deletion of one allele and a mutation of the other one. Redefining the current IHC model through adequate recognition of the p53 “low-level” phenotype as aberrant might be helpful in better understanding the prognostic role of p53 and possibly, in the future, correctly assigning target treatment.

**Abstract:**

Chromosomal instability (CIN) is very frequent in gastroesophageal adenocarcinoma (GEA) and it is characterized by *TP53* deletions/mutations resulting in p53 nuclear accumulation, as revealed by immunohistochemistry (IHC), which considers the cases with “high” staining levels to be positive. Aiming to improve aberrant *TP53* detection, droplet digital PCR (ddPCR) was used to evaluate *TP53* deletion in formalin-fixed, paraffin-embedded DNA (FFPE-DNA) and cell-free DNA (cfDNA). To further investigate the mutational *TP53* profile, next-generation sequencing (NGS) was performed in a subset of FFPE samples. After combining “low” and “high” IHC staining level groups, the proportion of deletion events was significantly higher compared to the “intermediate” group (72.9% vs. 47.5%, *p*-value = 0.002). The ddPCR *TP53* deletion assay was feasible for cfDNA but only had good agreement (72.7%, Cohen’s kappa = 0.48) with the assay performed with FFPE-DNA of the “low-level” group. NGS analysis confirmed that, in the “low-level” group, a high percentage (66.7%) of cases were aberrant, with disruptive mutations that probably led to p53 loss. Data suggested that p53 IHC alone underestimates the CIN phenotype in GEA and that molecular analysis in both solid and liquid biopsies could be integrated with it; in particular, in cases of completely negative staining.

## 1. Introduction

Adenocarcinomas of the gastroesophageal junction (GEJ) and stomach (GAC) rank fifth among the most common malignancies in the world and are the fourth leading cause of cancer-related death in both sexes worldwide. Esophageal adenocarcinoma (EAC), on the other hand, has a lower incidence, but it is still the sixth most common cause of cancer death globally, and it is characterized by increasing incidence, poor prognosis, and demanding treatment [1].

Despite the improvements in therapeutic options, the overall prognosis for patients with gastric-esophageal adenocarcinoma (GEA) remains poor, with a global 5 year survival rate lower than 30% for GAC and GEJ and of about 19% for EAC [2].

Recently, TCGA Research Network proposed to categorize GAC and GEJ into four molecular subgroups based on their predominant molecular profiles: Epstein–Barr virus-positive (EBV; 9%); microsatellite instability (MSI; 22%), characterized by mismatched repair (MMR) proteins deficiency; chromosomal instability (CIN; 50%), a phenotype featuring aberrant p53 expression; and genomic stability (GS; 20%), with normal MMR proteins expression and negative staining for EBV and p53 [3]. In addition, the overexpression/amplification of HER2/*ERBB2* has been found to be one of the most common (7–38%) genetic aberrations, especially in CIN tumors [3,4,5,6], and is included in diagnostic staining. EAC strongly resembles the CIN variant of GAC, suggesting that this cancer type could be considered a similar disease entity [7].

The subtype-specific genetic signature predicts the survival outcomes and benefits of adjuvant chemotherapy. EBV has the best prognosis in terms of relapse-free survival (RFS) and overall survival (OS). The GS subtype has the worst prognosis, while MSI has an intermediate one [8]. Furthermore, CIN seems to have an intermediate prognosis and, in addition, a better response to adjuvant chemotherapy; however, the prognostic and predictive roles of this phenotype are still debated [9].

*TP53* inactivation seems to be one of the main drivers for the genomic instability that characterizes the CIN subgroup. This gene, located on the 17th chromosome (17p13.1), encodes for a DNA-binding protein that regulates transcription and consists of two N-terminal transactivation domains followed by a conserved proline-rich domain, a central DNA-binding domain, and a C-terminus domain crucial for nuclear localization and oligomerization.

*TP53* acts as a tumor-suppressor gene through the regulation of several cellular functions, such as DNA damage response, cell cycle arrest, senescence, apoptosis, autophagy, and changes in the metabolism [10], and several mutations are associated with a variety of human cancers, including GEA (reviewed in [9]).

In the CIN subgroup, 71% of tumors harbor *TP53* mutations [3], which are mainly missense and occur in the region encoding the DNA-binding domain, leading to the abrogation of p53 degradation mediated by MDM2 binding. The lack of degradation results in excessive accumulation of p53 in the nucleus, while wild-type (wt) p53 protein is relatively unstable and has a short half-life.

Besides missense mutations, deletion of *TP53* is also a frequent event, reaching more than 70% in a pan-cancer analysis based on TCGA data [11]. Moreover, in this analysis, the most common configuration involved missense mutations in one allele with the deletion of the other, encompassing 41% of cases; however, a substantial proportion of tumors (31%) harbored the deletion together with an apparent wt allele. Hence, the frequency of deletion seems to exceed point mutations within the *TP53* gene [11].

Currently, due to the accumulation of mutated p53 in the cell nucleus, immunohistochemistry (IHC) staining of the protein represents the gold standard for the detection of aberrant p53. However, wt p53 can also be stabilized in response to various cellular stresses induced by DNA damage (reviewed in [10]). In addition, a lack of consensus still exists regarding the optimal cut-off for IHC to identify aberrant p53 [12,13,14,15,16,17,18,19,20,21].

Gonzalez et al. defined a cut-off of ≥70% positive tumor cells to consider the *TP53* phenotype as aberrant in GAC [22], showing that this cut-off could be used as a good surrogate for the interpretation of dysfunctional p53. On the other hand, relying on data from another study [12], researchers have estimated that 20% of p53 immunonegative gastric tumors have aberrant *TP53*, with nonsense and missense mutations equally represented. Others have reported discrepancies between IHC and mutation analysis, also showing aberrations in immunonegative cases with gastric [23] and ovarian cancer [24]. However, the majority of these studies in GAC sequenced *TP53* with Sanger sequencing, which reaches a sensitivity of 15–20%, and only analyzed gene mutations without considering the very frequent deletion event. It is only recently that Daun et al., through the more sensitive approach of next-generation sequencing (NGS), showed that p53 loss could be attributable to nonsense and frameshift mutations, but as in previous studies, these authors did not investigate the possible deletion of the other allele [25].

The mutational status of *TP53* has been investigated by NGS in various other cancer types, confirming that the majority of immunonegative cases carry genetic aberrations that are predicted to disrupt the amino acid sequence of the protein [26,27,28].

Thus, based on the observed existence of aberrant cases in the immunonegative p53 group and the fact that deletion is the most frequent event in tumors, whether accompanied by the mutation of the other allele or not, we hypothesized that *TP53* deletion/mutation analysis could be a valid method to estimate *TP53* status and to assign tumors/patients to the CIN subtype. For this purpose, we used droplet digital PCR (ddPCR) to analyze copy number variation (CNV) in terms of deletion of *TP53* in the solid biopsies of a retrospective GEA cohort and compared molecular results with IHC data. Moreover, to verify whether the application of ddPCR analysis in liquid biopsies could improve the detection of *TP53* deletion compared to solid biopsies alone, we also analyzed time-matched solid and liquid biopsies from a prospective GEA cohort.

In addition, we sequenced *TP53* using NGS in a subset of solid biopsies from both cohorts in order to evaluate the type and distribution of *TP53* mutations in GEA.

We believe that combining traditional IHC with very sensitive molecular techniques (ddPCR and NGS) could improve the classification of GEA into aberrant (CIN) or non-aberrant (GS) *TP53* tumors, helping in predicting the patient’s prognosis and response to adjuvant treatment.

## 2. Materials and Methods

### 2.1. Patients

For this study, FFPE samples from a retrospective cohort of 83 patients who underwent surgery between 2016 and 2019 were recovered from the archives of the IOV-IRCCS Pathology Unit. Moreover, 60 prospective patients referred to the Surgical Oncology of the Esophagus and Digestive Tract Unit of the Veneto Institute of Oncology (IOV-IRCCS, Padova, Italy) were enrolled between 2019 and 2020.

For the prospective cohort, in addition to the FFPE samples obtained from surgically resected specimens, blood sample (“time-matched” with the tissue) were collected just before surgery.

For both cohorts of patients, inclusion criteria were: (i) 18 years of age; (ii) histological diagnosis of GEA (all stages); and (iii) availability of FFPE tumor specimen from diagnosis and/or surgery resection. The exclusion criterion was concurrent diagnosis of synchronous or metachronous tumors within five years prior to the diagnosis of GEA.

The present study was approved by the IOV-IRCCS Comitato Etico per la Sperimentazione Clinica (CESC) (cod. number CESC IOV: 2019/72) and carried out in accordance with the Code of Ethics of the World Medical Association (Declaration of Helsinki and its later amendments). All the people involved in this study signed the informed consent form in accordance with the Helsinki Declaration.

### 2.2. Immunohistochemistry

In order to assess EBV, MSI, and CIN status in accordance with TCGA classification, FFPE samples from patients of the retrospective and prospective cohorts were tested for Epstein–Barr virus early RNA (EBER) antigen expression, the absence of mismatch repair (MMR) proteins (MLH1, MSH2, MSH6, and PMS2), and p53 aberrations, respectively.

Since HER2/*ERBB2* overexpression/amplification is one hallmark of GEA tumors, staining for this protein was also performed. EBER antigen testing, MMR proteins testing, and HER2/*ERBB2* staining were performed as previously described [29,30].

p53 IHC staining was performed on a Ventana Benchmark ULTRA platform (Roche, Monza, Italy) using an OptiView DAB IHC Detection Kit according to the manufacturer’s instructions. Briefly, 4 μM thick sections were deparaffinized and treated for antigen retrieval. After incubation with mouse monoclonal antibodies against p53 (DO-7; Roche Diagnostics Spa, Monza, Italia), tissue sections were treated with peroxidase inhibitor and buffers containing a cocktail of HQ-labeled antibodies and HRP-conjugated anti-HQ antibody. Then, slides were counterstained with Hematoxylin II. Each staining had internal positive and negative controls. p53 IHC evaluation was considered to indicate a positive phenotype when at least 70% of tumor cells disclosed strong nuclear immunostaining, defining the chromosomal instability (CIN) subtype group as previously described [22]. Each staining pattern was evaluated by a senior pathologist.

### 2.3. DNA Extraction

FFPE-DNA extraction was performed from eight 10 µm thick consecutive tumor specimen sections using the QIAamp Mini Kit or Micro Kit (QIAGEN, Milan, Italy) or the Maxwell RSC FFPE DNA kit (Promega, Milan, Italy) according to the instructions of the manufacturer. For tumor DNA analysis, a neoplastic component ≥70% was considered adequate; otherwise, a manual macro-dissection of tumor enrichment was performed. The quantity and the quality of DNA extracted were assessed using a NanoDrop 1000 spectrophotometer (Thermo Fisher Scientific, Milan, Italy).

Peripheral blood samples were collected in cell-free DNA BCT tubes (Streck, La Vista, NE, USA). Plasma was isolated from the blood corpuscular components by centrifugation at 2000× *g* for 10 min at 4 °C. Subsequent centrifugation at 16,000× *g* to remove cellular debris was performed and, finally, plasma was stored at −80 °C until cfDNA extraction.

**Figure A1:**
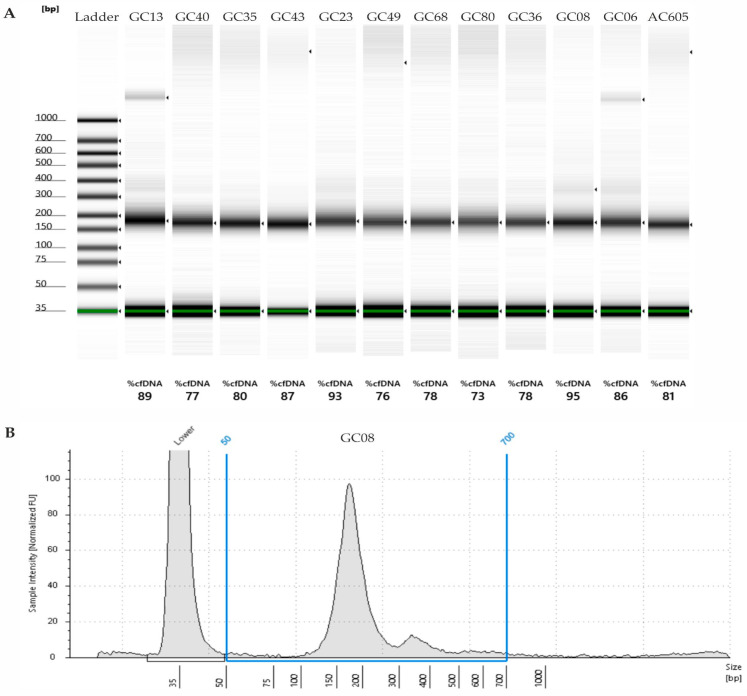
Quality assessment of cfDNA using Agilent Tape Station 2200 (cell-free DNA screen tape assay). Electrophoretic runs of ten representative samples (**A**). Electropherogram of a sample (GR08): the fragments between 150 and 200 bps represent the cfDNA (**B**).

cfDNA was extracted from 1 mL of plasma using the Maxwell RSC ccfDNA Plasma Kit (Promega, Milan, Italy) according to the manufacturer’s protocol, and concentration was measured using the fluorometric Qubit dsDNA HS Assay kit (Thermo Fisher Scientific, Milan, Italy); the quantity ranged between 6 and 30 ng/mL of plasma. The cfDNA quality was assessed with the Agilent Tape station 2200 using the cfDNA screen tape assay kit (Agilent Technologies, Milan, Italy). cfDNA with at least 70% purity was considered good quality and suitable for downstream analyses. A representative image of the quality of some cfDNA samples is shown in Figure A1 in Appendix A. Samples with germline DNA contamination were excluded from molecular analyses.

### 2.4. Droplet Digital PCR (ddPCR)

The presence of a deletion in *TP53* was evaluated by ddPCR in both FFPE-DNA and cfDNA using *EIF2C1* as reference, a diploid gene also known as Argonaute 1 (AGO1). The assays were run in duplicate.

The ddPCR reaction was carried out in a 20 µL volume mixture comprising 10 μL of 2× ddPCR Supermix for probes (no dUTP) (Bio-Rad, Milan, Italy), 1 µL of 20× target *TP53* primers/probe (FAM), and 1 µL of 20× reference *EIF2C1* primers/probe (HEX) (ddPCR™ Copy Number Variation Assays Validated, assay IDs: dHsaCP1000586 and dHsaCP2500349, respectively; Bio-Rad, Milan, Italy). The *TP53* primers/probe target the genomic region chr17:(7572961-7573083) mapping onto exon eight (hg19 genome reference). Each pair of primers/probes was used at a final concentration of 900 nM/250 nM. As the input of the DNA template, 15 ng/well for FFPE-DNA and 0.7 ng/well for cfDNA were used.

For droplet generation, 20 µL of the mixture and 70 µL of the droplet generator oil for probes were added into a DG8™ droplet generator cartridge, which was then loaded into a QX200 Droplet Generator (Bio-Rad, Milan, Italy). Then, 40 µL of the generated droplets were carefully transferred into a 96-well microplate, and PCR was performed using the following conditions: enzyme activation at 95 °C for 10′ followed by 40 cycles at 94 °C for 30″ (denaturation), 60 °C for 1′ (annealing and extension), and 98 °C for 10′ (enzyme inactivation). After PCR, the droplets were read with the QX200 droplet reader and analyzed with QuantaSoft™ version 1.7.4 software (Bio-Rad, Milan, Italy). The software generated copy number variation (CNV) values for each sample. To verify the assay accuracy, each ddPCR plate included a human-certified control DNA (CNV = 2) (Human Reference DNA #5190-4370) (Agilent Technologies, Santa Clara CA, USA).

To set up the cut-off value for considering *TP53* as deleted for the solid biopsy samples, FFPE-DNAs from normal mucosa of 24 retrospective and 19 prospective patients were analyzed. To set the cut-off for the liquid biopsy, 20 cfDNAs isolated from healthy volunteer plasma samples were analyzed. The mean CNV−2SD (standard deviation) value was considered for FFPE-DNAs and the mean −1 SD for cfDNA.

Samples with numbers of detected droplets <10,000 per 20 μL of PCR reaction were excluded from the analysis. When applicable, ddPCR experiments were designed, performed, and reported in line with the Digital MIQE Guidelines [31].

### 2.5. DNA Sequencing by Next Generation Sequencing (NGS)

*TP53* gene mutation analysis was performed using a KAPA HyperChoice customized Next Generation Sequencing (NGS) panel (Roche Diagnostic Spa, Monza, Italy).

Samples libraries were prepared from 300 ng of DNA input using the KAPA HyperCap FFPET DNA workflow v1.1 according to the manufacturer’s instructions (Roche Diagnostic Spa, Monza, Italy). The quantity and quality of the libraries were checked with a QuBit dsDNA HS Assay kit (Thermo Fisher Scientific, Milan, Italy) and a High Sensitivity D1000 ScreenTape Assay kit (Agilent Technologies, Milan, Italy), respectively. Enriched libraries were pooled and sequenced with NextSeq^TM^ 550 using a NextSeq 500/550 High Output Kit v2.5 (300 cycles) (Illumina, Milan, Italy).

Reads obtained were aligned to the reference genome and sequencing data were validated with JuliaOmix^TM^ software v2.21.0 (GenomeUp, Rome, Italy).

### 2.6. Statistics

All comparisons for categorical variables were performed using a chi-squared test. One-way analysis of variance (ANOVA) was performed to verify differences in the means of the distributions of NGS VAF between low/intermediate/high levels of IHC staining. The normality of the distributions was verified with standard tests, such as Shapiro–Wilk and Bartlett tests. All statistical analyses were performed with SAS (version 9.4). A *p*-value < 0.05 was considered statistically significant. Graphs were generated using GraphPad Prism software (version 9.2 for Windows, San Diego, CA, USA). The cartoon was generated using BioRender (https://www.biorender.com; accessed on 5 May 2023). Multiple graphs and images were arranged using CorelDRAW 2019 (64-bit).

## 3. Results

### 3.1. Clinicopathological Characteristics of Patients

#### 3.1.1. Retrospective Cohort

In order to assess *TP53* status, 83 retrospective FFPE samples were selected from the archives of the IOV-IRCCS Pathology Unit. The clinicopathological characteristics of the retrospective patients are summarized in Table 1. At diagnosis, the median age was 76 years (range: 44–97), with 50 males (60.2%) and 33 females (39.8%). Tumor stage I/II cases were less common compared to III/IV cases (41% vs. 59%, respectively). IHC analyses to define the molecular subtype revealed that 3.6% of samples were EBV+, 18.1% were MSI, 33.7% were CIN, and 45.8% were GS.

**Figure A2:**
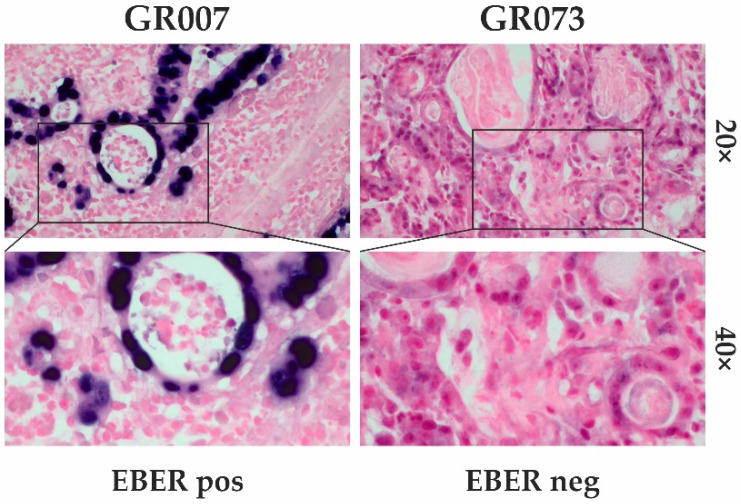
IHC staining for EBER antigen assessment to assign EBV positivity. Representative images of samples positive or negative for EBER antigen. In the positive sample, blue precipitate in the nucleus (IHC 20× and 40× magnification) represents the EBER antigen.

Representative images of MMR staining were reported in our previous publication focused on MSI status assessment [29]; an EBV staining example is shown in Figure A2.

#### 3.1.2. Prospective Cohort

To further assess the performance of the molecular methodologies in discriminating *TP53* status, 60 prospective patients were enrolled. For three patients, two sections were analyzed, resulting in a total of 63 FFPE samples. The median age was 69 years (range: 34–96), 26 patients had tumors at stage I/II (43.3%) and 34 at III/IV (56.7%). The clinicopathologic characteristics of this cohort were comparable to those of the retrospective cohort.

Regarding IHC typing, the most common group was GS (52.4%), followed by CIN (38.1%) and MSI (9.5%). None of the samples were EBV-positive (Table 2).

### 3.2. p53/TP53 Status Assessment by Droplet Digital PCR (ddPCR) in FFPE Samples

In order to verify the association between *TP53* deletion and IHC evaluation, we analyzed the FFPE-DNA samples from the retrospective cohort by ddPCR and compared the *TP53* CNV results with the p53 staining level.

Diagnostic IHC, performed and evaluated following Gonzalez et al. [22], showed that 55 samples (66.3%) were negative (<70% of positive nuclei), while 28 samples (33.7%) exhibited aberrant p53 staining (≥70% of positive nuclei) (Table 1).

**Figure 1:**
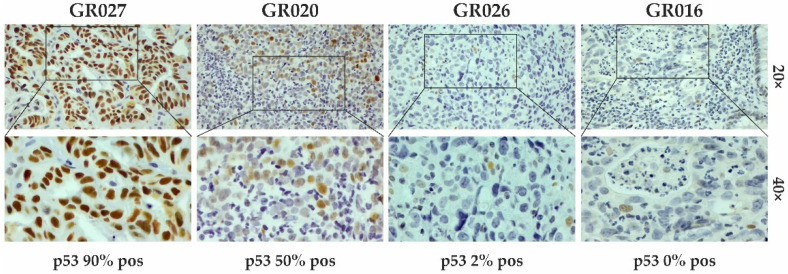
IHC staining for p53 evaluation. Representative images of samples with different percentages (%) of positive stained cells (brown color; IHC 20× and 40× magnification). In the positive sample, p53 staining was detected in 90% of cells (GR027), whereas the negative ones had percentages of positive cells lower than 70% (GR020, GR026, and GR016).

Representative IHC staining for p53 is reported in Figure 1.

*TP53* status was evaluated by ddPCR on the basis of the CNV value. The cut-off below which the samples were considered deleted was calculated as the CNV mean from 24 normal tissues −2 SDs (cut-off = 2.10).

In order to avoid biases due to the storage age of the FFPE samples, the 24 normal tissues were collected and processed from the same year range as the tumor tissues.

**Figure 2:**
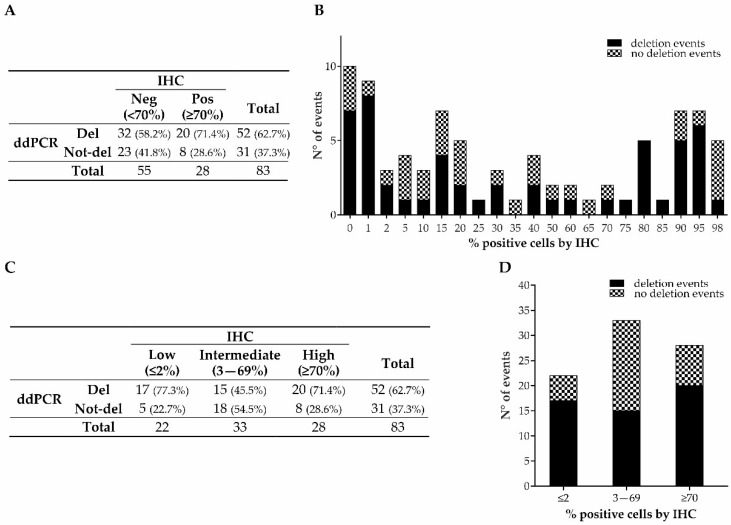
p53/*TP53* status, analyzed by IHC and ddPCR, of retrospective GEAs. Distribution of deleted and not-deleted cases in the groups categorized according to the IHC model based on Gonzalez et al. (**A**) and in the groups categorized as low, intermediate, and high percentages of IHC p53 staining (**C**). Distribution of deletion events by ddPCR in the whole cohort (**B**) and in the three newly categorized groups (≤2% (low), 3–69% (intermediate), and ≥70% (high)) (**D**).

Fifty-two FFPE samples (62.7%) showed that *TP53* had been deleted and thirty-one (37.3%) that it had not been deleted according to ddPCR analysis based on the selected cut-off (Figure 2A). Surprisingly, the number of *TP53*-deleted samples was almost double that of the IHC-positive samples (52 vs. 28; Figure 2A). Interestingly, the frequencies of deletion in the IHC-negative and -positive cases were similar (58.2% vs. 71.4%; *p*-value = 0.238). Consequently, there was an overall slight agreement between the IHC and ddPCR (Cohen’s kappa = 0.11). This result suggests that the current IHC interpretation could underestimate the number of aberrant p53 cases.

In order to investigate the reason for the discrepancy between the IHC and ddPCR, we looked at the distribution of deletion events in our cohort and noticed that, in the group considered negative by IHC (<70% positive nuclei), *TP53* was deleted with high frequency in the samples with low IHC staining (Figure 2B).

Based on this observation, we then categorized the samples into three groups based on the percentage of IHC staining: (i) low (≤2%), (ii) intermediate (3–69%), and (iii) high (≥70%). The frequencies of deleted samples in the low (77.3%) and high (71.4%) staining groups were significantly higher compared to the intermediate one (45.5%) (*p*-value = 0.029; Figure 2C,D).

These data suggest that samples exhibiting p53 IHC staining at low percentages (≤2%) should be considered as aberrant as samples with high IHC-positive staining (≥70%).

To verify this hypothesis, we categorized the samples by combining the ≤2% IHC-positive nuclei group together with the ≥70% IHC-positive nuclei group. The distribution of deletion events was significantly different between the ≤2% and ≥70% combined group and the 3–69% group (74% vs. 45.5%, respectively; *p*-value = 0.009; Table 3).

The ddPCR assay to analyze *TP53* deletion showed a sensitivity of 74% and a specificity of 54.55%, with a positive predictive value (PPV) of 71.15% and a negative predictive value (NPV) of 58.06%.

In addition, we extended ddPCR analysis to the prospective cohort. For this purpose, we analyzed the 63 FFPE samples from this cohort using ddPCR and calculated the cut-off to consider the sample deleted as the mean CNV from 19 normal FFPE samples −2 SDs (cut-off = 2.64). As in the retrospective cohort, the normal FFPE samples for these patients were also collected and processed from the same year range as the tumor tissues.

**Figure 3:**
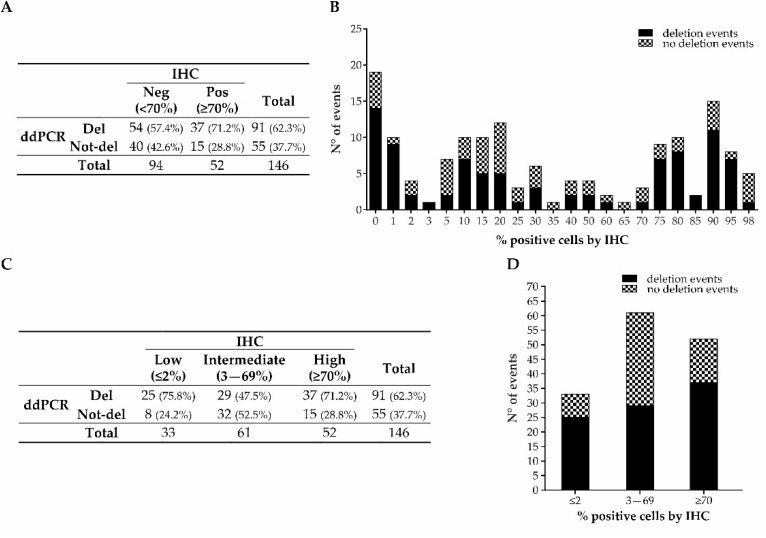
p53/*TP53* status, analyzed by IHC and ddPCR, of GEA prospective and retrospective combined cohorts. Distribution of deleted and not-deleted cases in the groups categorized according to the classic IHC model (**A**) and in the groups categorized according to the IHC staining level (**C**). Distribution of deletion events by ddPCR in the whole cohort (**B**) and in the three newly categorized groups (**D**).

By combining the ddPCR results obtained from the FFPE samples from the retrospective and prospective cohorts, the distribution of deleted cases considering the classical IHC-positive/negative categorization based on the 70% positive nuclei cut-off was confirmed, with 57.4% deleted cases in the <70% group and 71.2% in the ≥70% one (*p*-value = 0.102; Figure 3A,B). Again, when categorizing the cases into the three previously mentioned groups (≤2%; 3–69%; ≥70%), the distribution of deleted cases was significantly different (75.8%, 47.5%, and 71.2%, respectively; *p*-value = 0.007; Figure 3C,D).

Moreover, when combining the ≤2% and ≥70% groups together, the distribution of deletion events was confirmed to be significantly different compared to the 3–69% group (72.9% vs. 47.5%, respectively; *p*-value = 0.002; Table 4). The sensitivity, specificity, PPV, and NPV were similar to the retrospective samples alone (72.94%, 52.46%, 68.13%, and 58.18%, respectively).

### 3.3. TP53 Status Assessment by ddPCR in Cell-Free DNA (cfDNA) from the Prospective Cohort

In order to evaluate the feasibility of using the ddPCR assay to detect *TP53* deletion in liquid biopsies, cell-free DNA (cfDNA) isolated from the 60 prospective patients’ plasma samples was analyzed. To consider a sample *TP53*-deleted in cfDNA, a CNV cut-off of 2.58 was established by analyzing plasma samples from 20 healthy controls and using the CNV mean −1 SD. For the three patients with two available FFPE samples, in the case of discordant results between the solid and liquid biopsies, the FFPE specimen for which the deletion event was detected was considered as the “time-matched” sample, assuming that discordance was attributable to tumor heterogeneity.

Based on the concordance between the ddPCR results for the cfDNA and FFPE-DNA, samples were classified as: (i) deleted in both (“del/del”); (ii) not deleted in either (“not-del/not-del”); (iii) “del only in cfDNA”; or (iv) “del only in FFPE-DNA”.

**Figure 4:**
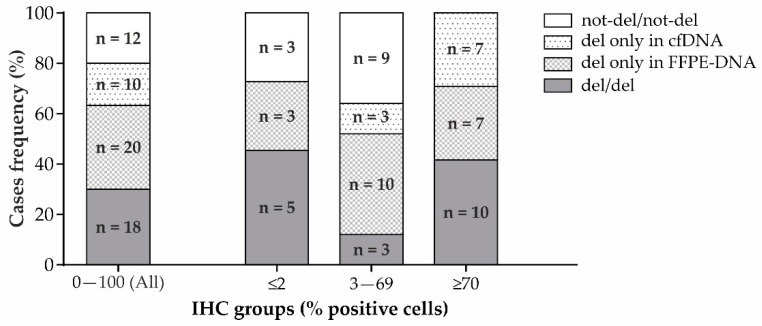
Concordant (del/del or not-del/not-del) and discordant (del only in cfDNA or del only in FFPE-DNA) results for ddPCR with FFPE-DNA and time-matched cfDNA from the prospective GEA patients’ cohort. Distribution of cases in the whole cohort (**left**) and in the three groups categorized according to the IHC staining level (**right**).

The frequency of deleted cases was 63.3% for FFPE-DNA and 46.7% for cfDNA, with a slight agreement between the two different biological matrices (50% agreement, Cohen’s kappa = 0.017). Indeed, while 18 cases were del/del and 12 were not-del/not-del, 30 cases were “del only in cfDNA” or “del only in FFPE-DNA” (Figure 4).

To better understand the reason for this great discordance, we analyzed also in this case the samples according to IHC staining levels: (i) low (≤2%), (ii) intermediate (3–69%), and (iii) high (≥70%). In the low IHC staining level group, five patients were “del/del” (5/11 = 45.4%) and three “not-del/not-del” (3/11 = 27.3%), while three were “del only in FFPE-DNA” (3/11 = 27.3%) (Figure 4). Globally, there was moderate concordance (72.7% agreement, Cohen’s kappa = 0.48). In the intermediate IHC staining level group, three patients were “del/del” (3/25 = 12%), nine were “not-del/not-del” (9/25 = 36%), ten were “del only in FFPE-DNA” (10/25 = 40%), and three were “del only in cfDNA” (3/25 = 12%) (Figure 4). There was no agreement between cfDNA and FFPE-DNA (48% agreement, Cohen’s kappa = −0.02). In the high IHC staining level group, ten patients were “del/del” (10/24 = 41.6%), zero were “not-del/not-del” (0/24 = 0%), seven were “del only in FFPE” (7/24 = 29.2%), and seven were “del only in cfDNA” (7/24 = 29.2%) (Figure 4). There was no agreement (41.66%, Cohen’s kappa = not computed due to there being zero cases in the “not-del/not-del” category).

### 3.4. Evaluation of TP53 Status by Next-Generation Sequencing (NGS) in FFPE Samples

Intrigued by the finding that the majority of FFPE samples categorized as having *TP53* deleted by ddPCR belonged to the IHC groups with ≤2% and >70% positive nuclei, we wondered if these two groups were also enriched in mutated *TP53*. To verify this hypothesis, a subgroup of retrospective and prospective FFPE samples (51 cases) were analyzed with an NGS custom panel designed to target the *TP53* gene. The mutations for each patient are reported in Table A1.

**Figure 5:**
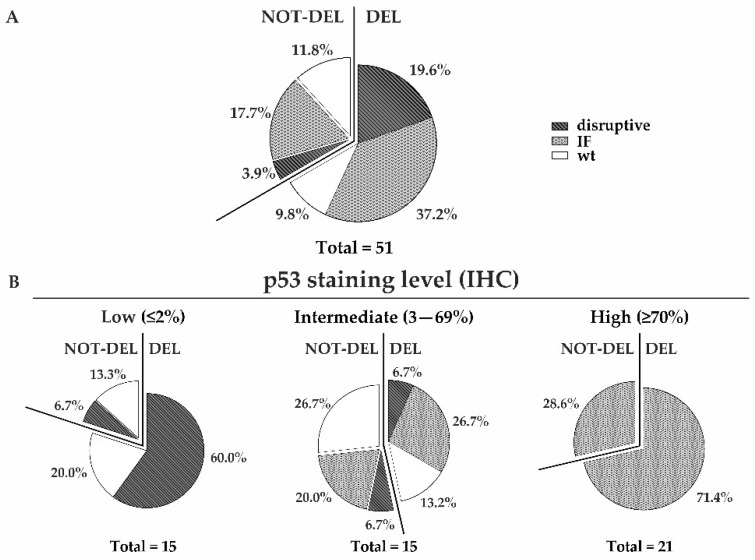
*TP53* status determined using a combined ddPCR and NGS analysis in a subset of retrospective and prospective FFPE samples. *TP53* mutation types (disruptive, inframe (IF)) in both deleted and not-deleted cases for the whole cohort (**A**) and for the three groups categorized according to the IHC staining level (**B**). The percentages of each mutation type are reported.

In our samples, we found nonsense or frameshift mutations that disrupted the amino acid sequence of the protein (disruptive mutations), as well as missense or indel mutations that did not alter the frame (inframe (IF) mutations). The overall mutation frequency was 78.4%, with disruptive and IF variants accounting for 23.5% and 54.9% of cases, respectively (Figure 5A).

Interestingly, of the 15 patients belonging to the ≤2% positive nuclei IHC group, 10 (66.7%) had disruptive mutations leading to probable mRNA decay or protein degradation. Nine of these ten patients (90%) had concurrent deletions of the other allele, showing that the majority of patients had probable two-hit events with the loss of one allele and the presence of a disruptive mutation in the other allele. Of the remaining five patients with *TP53* wt alleles in NGS, three (3/15 = 20%) showed *TP53* deletion by ddPCR, suggesting the loss of one allele and the retention of the wt one, while two (2/15 = 13.3%) cases showed no deletion by ddPCR, suggesting that the two alleles were both wt (Figure 5B).

In the 3–69% positive nuclei IHC group, the overall mutation frequency was 60% of cases, with the majority having IF mutations (7/15 = 46.7%). Four of these seven patients showed concurrent deletion of the other allele in the ddPCR. Two patients had disruptive mutations (2/15 = 13.3%) and six patients (6/15 = 40%) had at least one wt *TP53* allele with or without the deletion of the other allele (Figure 5B).

All the ≥70% positive nuclei IHC cases had IF mutations (21/21 = 100%). Fifteen patients (15/21 = 71.4%) also showed deletion of the other allele (Figure 5B).

Interestingly, the mean VAF for mutations was significantly different between the ≤2%, 3–69%, and ≥70% IHC-positive nuclei groups, with values of 0.30, 0.15, and 0.43, respectively (*p*-value = 0.002).

Considering the ddPCR and NGS data together, the *TP53* genotype was mainly constituted by one deleted allele and one carrying a disruptive mutation (del/disruptive) in the ≤2% group and by one deleted allele and one with an IF mutation (del/IF) in the ≥70% group. In the 3–69% group, there was a more heterogeneous scenario, with several different combinations of deleted and undeleted alleles accompanied or unaccompanied by different mutation types (Figure 5B).

## 4. Discussion

In this study, with the aim of better detecting *TP53* aberration and, consequently, identifying the CIN subtype in GEA patients, we used ddPCR to evaluate *TP53* deletion in FFPE-DNA samples. We then evaluated the possibility of transferring the ddPCR assay to cfDNA. Moreover, we evaluated the status of the remaining allele with NGS.

Traditionally, for GEA, as well as for other tumors, IHC staining of the protein p53 represents the “gold standard” for the evaluation of the aberrant status of the p53 pathway.

Many studies have faced the challenge of defining a positivity cut-off for the consideration of p53 status as aberrant, and a consensus regarding the interpretation has not yet been reached for several cancer types, including GEA [25,32,33]. In 2016, Gonzalez et al. defined 70% positive nuclei as the optimal IHC cut-off to define the CIN subtype, although with the awareness that, in low-percentage IHC staining, about 20% of cases could be aberrant and thus misclassified [22]. Indeed, Sanger sequencing found aberrant *TP53* in 20% of p53-negative gastric cancer cases, with nonsense and missense mutations equally represented [12].

Moreover, Schoop et al. also showed the existence of aberrant *TP53* in low-percentage IHC staining GEA cases. Indeed, these authors found that, after combining the extreme quartiles Q1 and Q4 of their patients’ cohort corresponding to low- and high-percentage p53 staining, respectively, there was a greater frequency of aberrant *TP53* compared to that for the intermediate quartiles Q2 and Q3 combined (63.4% vs. 36%, respectively) [23].

These studies in GEA, corroborated by similar results found for ovarian cancer [24], highlighted that low or complete lack of p53 IHC staining may be considered aberrant and that IHC alone underestimates the frequency of CIN cases. The reason for this misclassification could be that IHC is only a surrogate of the mutational status of a gene. Indeed, low p53 staining is not always associated with the normal turnover of a protein but could be the result of complete loss of the protein due to nonsense or frameshift mutations that lead to premature or altered stop codons in the coding sequence and consequent nonsense-mediated decay of mRNA or p53 ubiquitin-mediated proteasomal degradation, respectively [34,35].

Studies on *TP53* analysis in GEA have evaluated the presence of missense, nonsense, or indel mutations but have not investigated the presence of gene deletion. This event is very common, reaching more than 70% in a pan-cancer analysis based on TCGA data [11], and is also very frequent in GEA [36].

Due to the great frequency of *TP53* deletion events in GEA, in our study, we analyzed this alteration using ddPCR in both FFPE-DNA and cfDNA. Moreover, we further investigated the mutational status of the gene using NGS in a subset of FFPE-DNA.

Although, as stated above, previous studies have exclusively investigated *TP53* mutations, in agreement with them, we found that, in the low (≤2% positive cells) IHC staining group, several cases showed aberrant *TP53*. Indeed, in this group, the frequency of deleted samples was very high and similar to the high (≥70% positive cells) IHC staining group (77.3% vs. 71.4%), while the intermediate (3–69% positive cells) cases had lower frequencies of *TP53* deletion (45.5%). This finding was confirmed by combining the retrospective and prospective cohorts, revealing frequencies of deleted samples in the low, intermediate, and high IHC staining groups of 75.8%, 47.5%, and 71.2%, respectively.

These data suggest that p53 IHC staining alone underestimates CIN frequency in GEA and that low p53 levels should be considered aberrant as well as high levels. Indeed, after combining ≤2% and ≥70% cases together, the distribution of deletion events was significantly higher in this group than in the 3–69% one (72.9% vs. 47.5%, respectively).

In our study, we showed that performing the analysis in cfDNA was feasible. However, there was good agreement (72.7%) between the ddPCR in FFPE-DNA and cfDNA only in the low IHC staining group. In this group, both cfDNA and FFPE-DNA showed high percentages of deleted samples (45.4%).

In the intermediate IHC staining group, there was low agreement between the cfDNA and FFPE-DNA due to the abundant proportion of cases that were found to be deleted only with FFPE samples (40%). This result could be attributed to the relatively low abundance of clones harboring *TP53* deletion against the background of clones that did not have it. Against this heterogeneous background, the deletion signal in cfDNA could be diluted and, hence, not detected. On the other hand, intra-tumor heterogeneity, which was previously reported by other authors [37,38], could also explain the cases in which the deletion was found only in cfDNA.

Interestingly, in the high IHC level group, despite low concordance between cfDNA and FFPE-DNA, no cases were found to be not-deleted in either of the biological matrices, indicating that the deletion event could be considered highly probable.

Overall, it seems that liquid biopsy was informative for the low and high groups and not informative for the intermediate one. Considering that IHC alone seemed to be sufficient to successfully detect *TP53* aberration in the high group, we believe that liquid biopsy could be particularly useful in cases of low IHC staining. Moreover, in our opinion, the strong agreement between solid and liquid biopsies in the low group was due to the more homogeneous genetic status compared to intermediate and high groups.

This suggestion was confirmed by an NGS analysis of a subset of FFPE-DNA samples showing that, in the majority (90%) of aberrant low IHC staining cases, the main upstream molecular mechanism involved the deletion of one allele and the acquisition of a disruptive mutation in the other allele, as previously described using the two-hit mechanism (reviewed in [11]).

The two-hit event explains the almost complete absence of the protein revealed by IHC. The high mean VAF (0.30) for the disruptive mutations in the low IHC group suggested a high percentage of *TP53* mutated clones.

Intriguingly, 33.3% of the low IHC-positive nuclei cases had one wt allele, with or without the deletion of the other allele. Having one or both wild-type alleles together with a null or low percentage of staining would be compatible with the previous observation that wt p53 undergoes a physiological turnover that maintains the protein at a very low level, often undetectable by IHC (reviewed in [10]).

In the intermediate group, the main event was the occurrence of IF mutations with a moderate mean VAF (0.15) in one allele, often accompanied by deletion events in the other allele.

Interestingly, in all the patients from the high-level group, the main event was the occurrence of IF mutations with a very high mean VAF (0.43), which was also in this case, often accompanied by *TP53* deletion.

The difference in the VAF between intermediate- and high-level groups was in concordance with the more evident p53 accumulation detected by IHC in the latter group.

In the intermediate-level group, 40% had at least one wt *TP53* allele. Of those cases, some had a low p53 level, suggesting that the protein coded by the wt allele probably underwent normal turnover, while other cases showed moderate staining (15–25% positive nuclei), probably due to delayed degradation of the wt protein in response to cellular stress, as previously described (reviewed in [24]).

The overall mutation frequency found with NGS analysis was similar to those found in NGS pan-cancer and gastric cancer studies [11,25].

The NGS data confirmed that IHC staining alone underestimated CIN frequency in GEA, especially in the low-level IHC staining group.

A limitation of our study was the lack of data regarding NGS analysis in liquid biopsies. Indeed, it was not possible to perform this analysis due to the limited availability of plasma material. The majority of NGS panels, including ours, require starting from a minimum amount of 4 mL of plasma. Studying *TP53* mutational status with NGS could be interesting to verify if this approach could be applied as a further molecular approach to better detect aberrant p53 cases in GEA.

In our study, we did not find any associations between IHC p53 level and patients’ survival, probably due to the relatively limited number of cases. On the other hand, the association between a high/positive p53 level and poorer survival, as well as some clinicopathological features, has been demonstrated in previous studies but with some contradictory results. This scenario is probably due to the imperfection of the tools and the methodologies used to investigate *TP53*/p53 status, the availability of sufficient numbers of tumors for analysis, the heterogeneity of sample quality, and the great inter-laboratory variability in performing IHC in terms of the antibodies used and positivity cut-off chosen (reviewed in [9]). In this context, combining IHC with more sensitive molecular techniques and innovative approaches, such as liquid biopsies, in a large cohort of patients might be crucial to better understand the association between *TP53*/p53 status and prognosis.

Although CIN GEAs have exhibited the greatest response to adjuvant chemotherapy compared to GS cancer, they seem less responsive to immunotherapy. Recent studies on the CIN microenvironment showed fewer T and B cells and macrophage infiltrates, suggesting an immunosuppressive landscape [39].

Moreover, although new therapeutic implementations targeting aberrant *TP53* based on synthetic lethality, gene editing, and siRNA silencing are still at the preliminary stages [40], redefining the current IHC model through adequate recognition of the p53 low-level phenotype as aberrant might also be helpful in the future to correctly assign target treatment to CIN GEA patients.

## 5. Conclusions

Our study showed that the majority of low-level IHC staining cases are aberrant as well as the high-level ones, suggesting that traditional IHC should be matched with molecular techniques, such as ddPCR and NGS, which could make it possible to better investigate the aberrant phenotype at the genomic level, in particular when IHC is completely negative.

**Figure 6:**
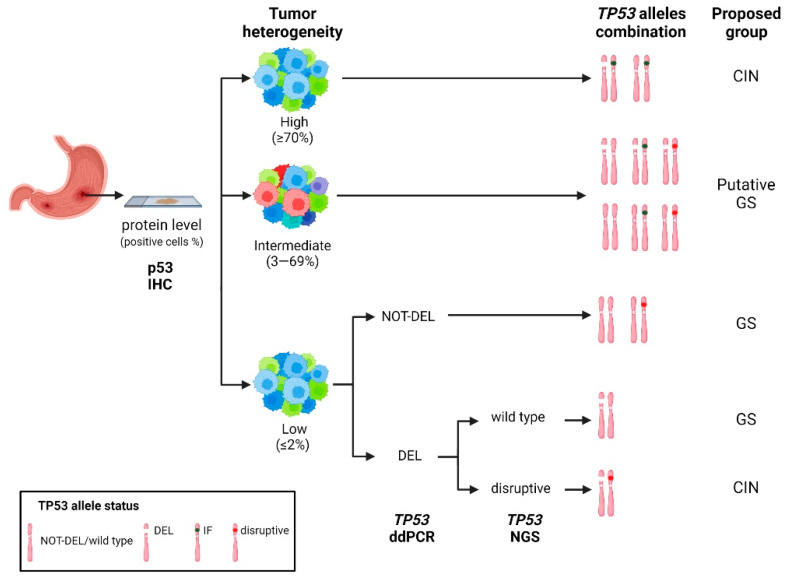
Hypothetical workflow based on a combination of traditional IHC and the high-throughput molecular techniques ddPCR and NGS to improve GEA classification for CIN/GS subtypes. IF, Inframe; CIN, chromosomal instability; GS, genomic stability.

Based on our ddPCR and NGS data, we propose a reclassification of the GEA subtypes with the inclusion of low IHC staining cases in the CIN subtype. The intermediate cases, with their more complex genomic profile and great tumor heterogeneity, should be considered as putatively GS until further investigations clarify their features (Figure 6).

In addition, regarding the use of liquid biopsies, ddPCR could be particularly useful to support the detection of aberrant cases in the low-level IHC group.

## Figures and Tables

**Table 1:** Clinicopathological characteristics of the retrospective cohort of gastroesophageal adenocarcinoma (GEA) patients.

	Retrospective Cohort
Patients	Total
	*N* (%) 83 (100)
Age	
Median (Q1; Q3)	76 (68; 81)
Range	44–97
Gender	
Male	50 (60.2)
Female	33 (39.8)
TNM stage *	
I/II	34 (41)
III/IV	49 (59)
IHC Typing	
EBV+	3 (3.6)
MSI ^#^	15 (18.1)
CIN ^#^	28 (33.7)
GS	38 (45.8)

Q1, first quartile; Q3, third quartile; IHC, immunohistochemistry; EBV, Epstein–Barr virus; MSI, microsatellite instability; CIN, chromosomal instability; GS, genomic stability; * clinical or pathological TNM was reported for inoperable or operable patients, respectively. ^#^ One patient was classified in both MSI and CIN subgroups due to the heterogeneity of the tumor.

**Table 2:** Clinicopathological characteristics of the prospective cohort of gastroesophageal adenocarcinoma (GEA) patients.

	Prospective Cohort
Patients	Total
	*N* (%) 60 (100)
Age	
Median (Q1; Q3)	69 (58.5; 76.5)
Range	(34–96)
Gender	
Male	41 (68.3)
Female	19 (31.7)
pTNM stage	
I/II	26 (43.3)
III/IV	34 (56.7)
IHC Typing *	
EBV+	0 (0)
MSI	6 (9.5)
CIN	24 (38.1)
GS	33 (52.4)

* IHC typing was performed with 63 FFPE sections because three patients had two available sections.

**Table 3:** Distribution of deletion events by ddPCR in the intermediate (3–69%) group and low and high (≤2% and ≥70%) combined group for the retrospective cohort.

		IHC		Total
		Intermediate (3–69%)	Low and High (≤2% and ≥70%)	
ddPCR	Del	15 (45.5%)	37 (74%)	52 (62.7%)
	Not-del	18 (54.5%)	13 (26%)	31 (37.3%)
	Total	33	50	83

ddPCR—droplet digital PCR; del—deleted; not-del—not deleted.

**Table 4:** Distribution of deletion events by ddPCR in the intermediate (3–69%) group and low and high (≤2% and ≥70%) combined group for the prospective and retrospective cohorts combined.

		IHC		Total
		Intermediate (3–69%)	Low and High (≤2% and ≥70%)	
ddPCR	Del	29 (47.5%)	62 (72.9%)	91 (62.3%)
	Not-del	32 (52.5%)	23 (27.1%)	55 (37.7%)
	Total	61	85	146

ddPCR—droplet digital PCR; del—deleted; not-del—not deleted.

## Data Availability

The data presented in this study are available from the corresponding author on request.

## Appendix A

**Table A1:** *TP53* status determined by ddPCR and NGS analyses in a subset of retrospective and prospective FFPE samples.

	Sample	IHC (%)	ddPCR FFPE-DNA *	NGS FFPE-DNA				ddPCR/NGS Combined Data
				Sequence Change	Exon	VAF	Varsome Prediction	
1	GR001	0	Del	c.104del/p.Leu35CysfsTer9	4/11	0.1917	Pathogenic	Del/disruptive
2	GR004	0	Not-del	c.637C>T/p.Arg213Ter	6/11	0.1918	Pathogenic	Not-del/disruptive
				c.524G>A/p.Arg175His	5/11	0.0143	Pathogenic	
3	GR008	0	Del	c.158G>A/p.Trp53Ter	4/11	0.4982	Pathogenic	Del/disruptive
4	GR016	0	Del	c.830del/p.Cys277PhefsTer68	8/11	0.0769	Likely pathogenic	Del/disruptive
5	GR022	0	Del	c.916C>T/p.Arg306Ter	8/11	0.4483	Pathogenic	Del/disruptive
6	GR028	0	Del	c.159G>A/p.Trp53Ter	4/11	0.3838	Pathogenic	Del/disruptive
				c.161dup/p.Thr55HisfsTer2	4/11	0.3810	Likely pathogenic	
7	GR034	0	Del	c.949C>T/p.Gln317Ter	9/11	0.2632	Pathogenic	Del/disruptive
				c.587G>A/p.Arg196Gln	6/11	0.0121	Pathogenic	
8	GR050	0	Del	c.724_731del/p.Cys242ArgfsTer19	7/11	0.3852	Likely pathogenic	Del/disruptive
9	GR062	0	Not-del	wt	-	-	-	Not-del/wt
10	G016	0	Del	c.637C>T/p.Arg213Ter	6/11	0.2537	Pathogenic	Del/disruptive
11	G036	0	Del	wt	-	-	-	Del/wt
12	G069	0	Del	c.326del/p.Phe109SerfsTer14	4/11	0.3125	Likely pathogenic	Del/disruptive
				c.532C>G/p.His178Asp	5/11	0.0455	Pathogenic	
				c.476C>G/p.Ala159Gly	5/11	0.0455	Pathogenic	
13	GR010	1	Del	wt	-	-	-	Del/wt
14	GR049	2	Del	wt	-	-	-	Del/wt
15	G043	2	Not-del	wt	-	-	-	Not-del/wt
1	G048	5	Not-del	wt	-	-	-	Not-del/wt
2	G049	5	Del	c.1040C>G/p.Ala347Gly	10/11	0.0909	VUS	Del/IF
3	GR061	10	Not-del	c.799C>T/p.Arg267Trp	8/11	0.1754	Pathogenic	Not-del/IF
4	G070	10	Del	c.706T>C/p.Tyr236His	7/11	0.1111	Pathogenic	Del/IF
				c.542G>A/p.Arg181His	5/11	0.1000	Pathogenic	
5	GR029	15	Del	c.1146del/p.Lys382AsnfsTer40	11/11	0.2198	Pathogenic	Del/disruptive
6	G077	15	Del	wt	-	-	-	Del/wt
7	GR033	20	Not-del	wt	-	-	-	Not-del/wt
8	G020	20	Del	wt	-	-	-	Del/wt
9	G039 N5	20	Not-del	c.902del/p.Pro301GlnfsTer44	8/11	0.0135	Pathogenic	Not-del/disruptive
10	G068	20	Del	c.524G>A/p.Arg175His	5/11	0.4552	Pathogenic	Del/IF
11	G021	25	Not-del	wt	-	-	-	Not-del/wt
12	G074	25	Not-del	wt	-	-	-	Not-del/wt
13	G075	30	Not-del	c.652_654dup/p.Val218dup	6/11	0.0476	Pathogenic	Not-del/IF
14	GR002	40	Not-del	c.856G>A/p.Glu286Lys	8/11	0.1479	Pathogenic	Not-del/IF
				c.772G>T/p.Glu258Ter	7/11	0.0118	Pathogenic	
				c.400T>C/p.Phe134Leu	5/11	0.0784	Pathogenic	
15	GR043	60	Del	c.713G>A/p.Cys238Tyr	7/11	0.0633	Pathogenic	Del/IF
1	A564	75	Del	c.326T>G/p.Phe109Cys	4/11	0.6904	Pathogenic	Del/IF
2	G002	75	Not-del	c.818G>A/p.Arg273His	8/11	0.2280	Pathogenic	Not-del/IF
3	G064	75	Del	c.1024C>G/p.Arg342Gly	10/11	0.7143	VUS	Del/IF
				c.380C>T/p.Ser127Phe	5/11	0.4000	Pathogenic	
				c.310del/p.Gln104ArgfsTer19	4/11	0.0417	Pathogenic	
4	G045	75	Not-del	c.524G>A/p.Arg175His	5/11	0.4444	Pathogenic	Not-del/IF
				c.482C>G/p.Ala161Gly	5/11	0.1000	Likely pathogenic	
				c.532C>T/p.His178Tyr	5/11	0.0909	Pathogenic	
5	G072	75	Del	c.455C>T/p.Pro152Leu	5/11	0.0580	Pathogenic	Del/IF
6	A577	80	Del	c.451C>T/p.Pro151Ser	5/11	0.6603	Pathogenic	Del/IF
7	A573	80	Del	c.703_705del/p.Asn235del	7/11	0.1329	Pathogenic	Del/IF
8	G024	85	Del	c.770T>A/p.Leu257Gln	7/11	0.5581	Pathogenic	Del/IF
				c.950A>G/p.Gln317Arg	9/11	0.0321	VUS	
9	GR037	90	Del	c.623A>T/p.Asp208Val	6/11	0.4687	Likely pathogenic	Del/IF
10	GR054	90	Del	c.700T>G/p.Tyr234Asp	7/11	0.7577	Pathogenic	Del/IF
11	GR075	90	Del	c.524G>A/p.Arg175His	5/11	0.1948	Pathogenic	Del/IF
12	G013	90	Not-del	c.809T>C/p.Phe270Ser	8/11	0.6015	Pathogenic	Not-del/IF
13	G025	90	Del	c.817C>T/p.Arg273Cys	8/11	0.0900	Pathogenic	Del/IF
14	G039 N10	90	Del	c.733G>A/p.Gly245Ser	7/11	0.5567	Pathogenic	Del/IF
15	G042	90	Del	c.808T>G/p.Phe270Val	8/11	0.6376	Pathogenic	Del/IF
16	G050	90	Not-del	c.528C>G/p.Cys176Trp	5/11	0.1842	Pathogenic	Not-del/IF
17	G054	90	Del	c.638G>T/p.Arg213Leu	6/11	0.5130	Pathogenic	Del/IF
18	GR084	95	Not-del	c.722C>T/p.Ser241Phe	7/11	0.4765	Pathogenic	Not-del/IF
19	GR063	98	Del	c.818G>A/p.Arg273His	8/11	0.2513	Pathogenic	Del/IF
20	GR078	98	Del	c.638G>A/p.Arg213Gln	6/11	0.3137	Pathogenic	Del/IF
21	GR086	98	Not-del	c.742C>T/p.Arg248Trp	7/11	0.4335	Pathogenic	Not-del/IF

* ddPCR assay maps in exon eight targeting a region between the positions 17:7669643 and 7669765, as declared by the producer and according to hg19 reference genome. IHC—immunohistochemistry; FFPE—formalin-fixed, paraffin-embedded; NGS—next-generation sequencing; ddPCR—droplet digital PCR; VAF—variant allele frequency; del—deleted; IF—inframe; wt—wild-type; VUS—variant of uncertain significance.
